# The Relationship between Acceptance and Sleep–Wake Quality before, during, and after the First Italian COVID-19 Lockdown

**DOI:** 10.3390/clockssleep4010016

**Published:** 2022-03-07

**Authors:** Marco Fabbri, Luca Simione, Monica Martoni, Marco Mirolli

**Affiliations:** 1 Department of Psychology, University of Campania Luigi Vanvitelli, Viale Ellittico 31, 81100 Caserta, Italy; 2 Institute of Cognitive Sciences and Technologies, National Research Council (ISTC-CNR), Via San Martino della Battaglia 44, 00185 Rome, Italy; luca.simione@istc.cnr.it (L.S.); marco.mirolli@istc.cnr.it (M.M.); 3 Department of Experimental, Diagnostic and Specialized Medicine, St.Orsola-Malpighi Hospital, University of Bologna, Via Massarenti 9, 40138 Bologna, Italy; monica.martoni@unibo.it

**Keywords:** sleep quality, mindfulness, COVID-19 lockdown, distress, acceptance, anxiety, path analysis

## Abstract

Several studies have reported that the COVID-19 pandemic has had deleterious effects on sleep quality and mood, but the mechanisms underlying these effects are not clearly understood. Recently, it has been shown that the acceptance component of mindfulness reduces anxiety, and, in turn, lower anxiety improves sleep quality. The purpose of this cross-sectional study was to assess changes in mindfulness traits, sleep–wake quality, and general distress, before, during, and after the first COVID-19 wave, testing the model in which acceptance influences sleep through anxiety in each period. A total of 250 participants were recruited before (Pre-Lockdown group: 69 participants, 29 females, 33.04 ± 12.94 years), during (Lockdown group: 78 participants, 59 females, 29.174 ± 8.50 years), and after (After-Lockdown group: 103 participants, 86 females, 30.29 ± 9.46 years) the first Italian lockdown. In each group, self-report questionnaires, assessing mindfulness facets, distress, and sleep–wake quality, were administered and assessed. The Lockdown group reported lower acceptance and higher depression, while the After-Lockdown group reported lower sleep–wake quality and higher anxiety. The results of the path analysis confirmed that higher acceptance reduced anxiety and higher anxiety decreased sleep–wake quality in all groups. Our results confirm that acceptance influences sleep through the mediating role of anxiety.

## 1. Introduction

Sleep is a vital process for maintaining the quality of human life. Adequate sleep quality and duration are essential for life, health, and well-being, whereas sleep deprivation has a strong negative impact on daily behavior and mental health [1]. For example, sleep disorders can be symptoms of a disease or predictors of a future disease, such as depression [2]. Interestingly, it has been reported that the spread of the coronavirus disease in 2019 (known as COVID-19) showed wide-ranging disruptions to personal schedules, psychological health, and sleep, throughout the world [3,4]. In reaction to the COVID-19 pandemic, the Italian Government implemented a total lockdown during the first wave of the contagion (from 10 March to 3 May 2020); this involved home confinement, social distancing for the entire population, and the closure of most business activities. These measures affected both sleep and mental health, resulting in an increase in sleep difficulties, especially in people with higher levels of depression, anxiety, and stress [5].

A second contagion outbreak occurred in Italy from 14 September to 31 December 2020, and the government adopted new measures to control virus propagation. During this period, two studies confirmed sleep disturbances, a prevalence of depressive symptomatology, and increased perceived stress, similar to the first lockdown [6,7]. However, in between the two lockdowns, improvements in subjective sleep quality, sleep latency, and sleep disturbances were observed [6]. For example, Conte et al. [7] indicated that the alterations to sleep (e.g., sleep timing) that occurred during the first lockdown returned toward baseline, as demonstrated by the values measured during the month preceding the second lockdown.

These findings were further confirmed during the third lockdown that occurred in Italy the following spring (from 15 March to 21 June 2021). On one hand, Conte et al. [8] demonstrated the presence of disrupted sleep and increased poor subjective sleep, similar to what had been reported in the previous waves [3,4,5,6,7]. On the other hand, the authors also described that sleep duration was in the normal range, suggesting that the (repetitive) experience of lockdown mainly affected the quality rather than the quantity of sleep. In a larger sample (N = 339), in Hong Kong, Lam et al. [9] reported similar percentages of insomnia symptoms between the second and third waves of the outbreak, suggesting that sleep problems were common during the pandemic. The prevalence of anxiety and depression symptoms was also similar during the two waves [9]. Hence, while all these studies clearly demonstrated a worsening of sleep quality and mood during the COVID-19 lockdown, it is not clear whether an improvement in sleep quality and mood (i.e., a return toward baseline values) occurs after lockdowns. Furthermore, it is important to note that these studies, investigating the evolution of sleep quality and mental health through the waves of the COVID-19 contagion, did not truly assess the selected variables during different time periods (i.e., before, during and/or after the various lockdowns), but, rather, compared the variables assessed at one point in time (e.g., during a lockdown) with retrospective reports taken concurrently at the same point in time [3,6,7,8,9]. For this reason, the reliability of these findings is dubious.

Few studies have investigated sleep quality and mood at the end of a COVID-19 lockdown. For example, a longitudinal study in France compared sleep disorders during the first week, in the middle, and at the end of confinement, as well as one month after the end [10]. The prevalence of sleep problems decreased during the last weeks of lockdown, and this trend was further confirmed in the follow-up assessment, with an improvement in sleep quality in 25% of participants. Similarly, Waage et al. [11] reported that more than 80% of Norwegian nurses did not show any change in sleep duration or sleep quality after the first wave of the COVID-19 pandemic compared to before (data collected between June and September 2020). These studies may indicate that the distress determined by lockdown (home confinement, social distancing, closure of activities) probably affected the sleep–wake cycle immediately, with a decrease in sleep quality and an increase in sleep problems. This influence, however, seems to be temporary, given that after lockdowns, sleep impairment seems to be naturally resolved, probably thanks to a reduction in general distress. However, the amount of time that is needed for this return to baseline is not clear, given that the two studies covered different time windows (e.g., from May to June for [10] and from June to September for [11]). Furthermore, and more importantly, much remains to be understood about which psychological factors are responsible for a reduction in distress symptoms and, consequently, for a “return-to-baseline” of sleep–wake problems.

In the past decade, it has been reported that mindfulness, defined as being present in the moment, intentionally and with a nonjudging attitude [12], is associated with better sleep quality [13] and lower levels of depression and anxiety [14]. In particular, Simione et al. [13] reported that mindfulness had a positive effect on sleep quality, which was mediated by stress. However, mindfulness is a multidimensional concept [12,15], and different relationships between mindfulness components and different outcomes have been proposed. In particular, Lindsay and Creswell [16] proposed the Monitoring and Acceptance theory (or MAT), according to which, mindfulness works mainly through attention monitoring and acceptance. Attention monitoring (or monitoring, for short) is defined as the ability to notice what is happening in one’s internal and external environment during the present moment, while acceptance is defined as an open, receptive, and nonjudgmental attitude toward one’s own experiences. Specifically, the theory proposes that monitoring improves cognitive function but also increases affective reactivity, while monitoring and acceptance together lead to decreased stress and increased psychophysical well-being [16]. However, Simione et al. [17] challenged this theory, by demonstrating that acceptance was the only predictor of several benefits of mindfulness, including those regarding sleep quality. Interestingly, Mirolli et al. [5] recently confirmed these findings in a longitudinal study by comparing mindfulness traits, general distress, and sleep quality, before and during the first Italian lockdown. In particular, this study demonstrated that acceptance (and not monitoring), influenced sleep through the mediating role of anxiety [5,17]. Hence, the acceptance component of mindfulness could be one of the main psychological variables accountable for a reduction in distress and, in turn, in sleep–wake problems.

The present research aims to assess the relationships between mindfulness (in particular, its acceptance and monitoring components), distress symptoms, and sleep–wake quality, in a cross-sectional study, involving measurements taken before, during, and after the first Italian lockdown. Considering the literature presented above, we expected two main findings to emerge: (a) compared to the other two phases, the lockdown phase would involve lower acceptance, higher distress, and worsened sleep quality; (b) the model according to which acceptance influences sleep quality through the mediating role of distress [5,13] would persist during all three phases (before, during, and after lockdown).

## 2. Results

### 2.1. Effects of Lockdown on Mindfulness, Well-Being, and Sleep

Table 1 reports the means and standard deviations of each variable in each phase, along with the statistics of the corresponding ANCOVA and relative post-hoc comparisons. A group effect was observed on all FFMQ subscales, except for describing. In particular, observing was higher in the Lockdown group than in the Pre-Lockdown group; acting with awareness was higher in the Pre-Lockdown group than in the After-Lockdown group; nonjudging was higher in the Pre-Lockdown group than in both the Lockdown and After-Lockdown groups; nonreacting was lower in the After-Lockdown group than in both the Pre-Lockdown and Lockdown groups. Acceptance (calculated as the sum of nonjudging and nonreacting) was higher in the Pre-Lockdown than in the After-Lockdown group. We did not find any significant group effect on the HADS total score, while we observed a significant effect on both the anxiety and depression scores: in particular, anxiety was higher in the After-Lockdown group than in the Pre-Lockdown group, while depression was lower in the After-Lockdown group than in both the Pre-Lockdown and Lockdown groups. Finally, we found group effects for the total MSQ score and for both its sleep and wake factors, separately. Specifically, the post-hoc test revealed the same pattern for all three measures: all these scores increased from Pre-Lockdown to Lockdown and from Lockdown to After-Lockdown. As stated in Section 4.3, we controlled for the rMEQ score, for which no significant group effect was found.

### 2.2. Anxiety Mediates the Effect of Acceptance on Sleep–Wake Problems

**Figure 1:**
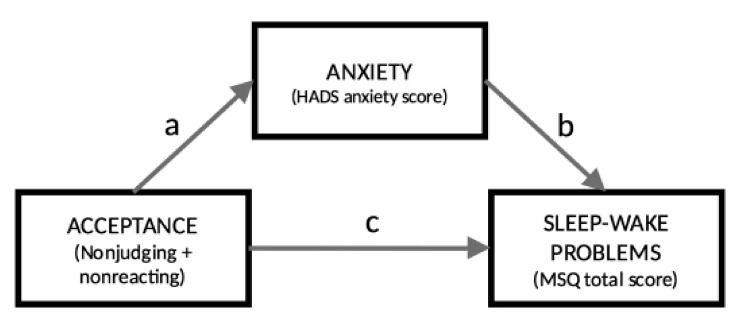
The tested mediation model.

We tested the model, according to which, acceptance influences sleep–wake problems through the mediational role of anxiety (Figure 1). First, we conducted a configural invariance model, in which the same model was separately estimated for each group. The model revealed acceptable fit statistics, *χ^2^*(3) = 4.59, *p* = 0.20, CFI = 0.99, TLI = 0.99, RMSEA = 0.08, CI_RMSEA_ = [0.01, 0.21], and SRMR = 0.02. The coefficients are reported in Table 2, separately for each group. As shown in Table 2, in each group, the path from acceptance to anxiety (*a* parameter) had a significant negative association. We found a significant positive association from anxiety to sleep–wake problems (*b* parameter), except for the After-Lockdown group, in which the path tended toward significance (*p* = 0.10). The direct path from acceptance to sleep–wake problems (*c* parameter) was not significant in the Pre-Lockdown and Lockdown groups, whereas it was significant and negative in the After-Lockdown group. The indirect effect from acceptance to sleep–wake problems, mediated by anxiety, was significant and negative for the Pre-Lockdown and Lockdown groups, whereas it showed a significant trend only in the After-Lockdown group. Finally, the total effect of acceptance on anxiety (direct + indirect effects) was significant in all groups.

**Figure 2:**
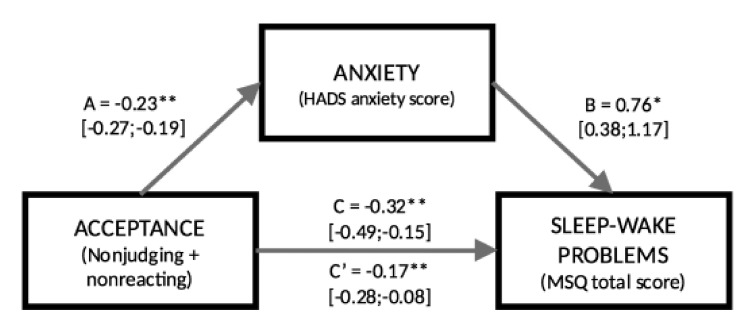
The metric invariance model with all estimated parameters. * *p* < 0.05, ** *p* < 0.01.

We then tested the metric invariance model, i.e., a model in which we constrained all fitted parameters to be equal across groups (Figure 2). This model showed adequate fitting statistics, with *χ^2^*(9) = 19.15, *p* = 0.06, CFI = 0.96, TLI = 0.95, RMSEA = 0.09, CI_RMSEA_ = [0.01, 0.16], and SRMR = 0.06. In this constrained model, all the tested paths were significant (Table 3), from acceptance to anxiety, from anxiety to sleep–wake quality, and from acceptance to sleep–wake quality. Moreover, the indirect effect of acceptance on sleep–wake quality was also significant, indicating that a partial mediation model fitted the data well in all phases. To test metric invariance, we compared its fit statistics to the configural invariance model. The test revealed a non-significant difference, with *Δχ^2^* = 11.86, df = 6, *p* = 0.07, suggesting that the constrained model could be considered adequate for all phases. Given that the previous comparison, however, reported a tendency toward significance, we inspected the constrained parameters in the model to assess which one should be released, to improve the fit for the metric invariance model. This analysis revealed that the *c* parameter (direct path from acceptance to sleep–wake quality) had a significant impact on the model fit when constrained. Thus, we fitted a new model, in which we freely estimated this parameter for each group. This model revealed improved fitting statistics compared to the fully constrained model, *χ^2^*(9) = 13.04, *p* = 0.16, CFI = 0.98, TLI = 0.96, RMSEA = 0.07, CI_RMSEA_ = [0.01, 0.15], and SRMR = 0.05. Table 3 also reports the *c* parameter estimated for each group: the direct path was not significant in the Pre-Lockdown group, nearly significant in the Lockdown group (*p* < 0.10), and significant in the After-Lockdown group (*p* < 0.01). Hence, the effect of acceptance on sleep–wake quality was fully mediated by anxiety in the Pre-Lockdown and Lockdown groups, while it was only partially mediated in the After-Lockdown group. The comparison between this model with a freely estimated *c* parameter and the configural invariance model showed a non-significant difference, with *Δχ^2^* = 8.45, df = 6, *p* = 0.21.

## 3. Discussion

The aim of the present research was twofold: first, to assess dispositional mindfulness, perceived distress, and sleep–wake quality in three different groups, before, during, and after the first Italian lockdown; second, to test the reliability of the model reported in [5], according to which, acceptance affects anxiety, which, in turn, affects sleep–wake quality, independently from the period in which these variables were measured.

The results of the ANCOVAs showed that lockdown impacted dispositional mindfulness facets differently. In line with [5], during lockdown, we observed an increase in attention monitoring (measured as observing), which tended to return toward baseline levels in the After-Lockdown group, and a decrease in acceptance (measured either as nonjudging and nonreacting or as their sum), which, on the contrary, continued to decrease, even after the end of the lockdown period. The temporary increase in monitoring could be explained by the fact that participants in the Lockdown group were more vigilant (more monitoring) for the threat of illness, while the end of the lockdown could have led to this vigilance decreasing toward baseline values. On the other hand, the same heightened perceived risk could have reduced acceptance because the judgment of one’s thoughts and a high reactivity were considered to be important, in order to protect one’s own safety. The fact that acceptance did not return to baseline after the end of lockdown is particularly interesting. From the point of view of mindfulness dynamics, it suggests that acceptance is a vulnerable skill, which, once diminished, is difficult to regain. Furthermore, given the association between acceptance and many beneficial outcomes, including lower levels of stress, depression, anxiety, worry, rumination, and sleep problems, and a higher level of well-being [17,18,19,20,21], the lack of a return to baseline of acceptance after lockdown may be related to the negative long-term psychological consequences of the COVID-19 pandemic (e.g., [22]). This seems to be confirmed by our results on anxiety and sleep–wake quality, which, indeed, increased from the Pre-Lockdown group to the After-Lockdown group (see below).

The hypothesis that the worsening of sleep–wake quality due to lockdown depended on a decrease in acceptance with the mediation of anxiety [5] was strongly confirmed by the path analysis models on the three samples. As shown by the metric invariance model, acceptance influenced sleep–wake quality, both directly and indirectly, through the mediation of anxiety. Several scholars have already proposed that mindfulness can be beneficial for insomnia and sleep problems. Shallcross et al. [23] proposed that mindfulness improves sleep through the mechanisms of experiential awareness, attentional control (both related to monitoring), and acceptance, which collectively contribute to disturbances, such as rumination, attentional bias, and distorted perceptions regarding one’s own sleep impairments. Indeed, an association has been demonstrated between acceptance and a reduced level of worry and rumination [21], and between worry/rumination and anxiety [24]. In line with previous research [5,17], our results support the view that it is the acceptance component of mindfulness alone (and not monitoring) that is responsible for its beneficial effects on sleep. Furthermore, our findings are also in line with the recent metacognitive model of insomnia [15], according to which, mindfulness benefits insomnia by reducing the distress induced by worries related to sleep (which was also supported by [13]). Our present findings are particularly important given that the same model was confirmed independently from the period (and in different participants), in which acceptance, anxiety, and sleep–wake quality were measured, suggesting that acceptance can be considered as a dispositional (internal) resource for coping with general distress and, hence, with sleep–wake problems. Thus, our results support the recommendation to use mindfulness-based interventions for treating insomnia and sleep-related disturbances [15,23], as well as the suggestion to design mindfulness-based interventions, particularly focused on developing acceptance in order to prevent sleep–wake problems, especially in stressful situations, such as the COVID-19 pandemic [5].

Regarding sleep and wake factors, we found that the Lockdown group reported poor sleep and wake quality compared to the Pre-Lockdown group, confirming the results of different studies showing the deleterious impact of lockdowns during different waves in different periods of the year [3,5,6,7,8,9]. However, in contrast with previous studies [10,11], we found that the After-Lockdown group reported even higher sleep–wake disturbances than the Lockdown group. This discrepancy could be explained by the different methodology adopted. In particular, in the present study, we assessed sleep–wake problems through a questionnaire (i.e., MSQ) with good psychometric properties [25], while the other studies used ad-hoc questions. Indeed, the MSQ required participants to report sleep and wake difficulties during the past seven days, whereas Beck et al. [10] and Waage et al. [11] asked participants to rate their sleep–wake quality with specific questions, such as “*After the pandemic hit Norway, I sleep (1) much poorer than before, (2) to some degree poorer that before, (3) no change, (4) to some degree better than before and (5) much better than before*”. This different method could explain why we did not find a “return to baseline” level for sleep–wake quality. Moreover, the different period (and context) in which the studies were performed could also explain why we found a higher level of sleep–wake problems in the After-lockdown group. Indeed, the study performed in France [10] was conducted across four different periods, with only a few days designated for individuals to participate in the survey (from March 31 to April 2, from April 15 to April 17, from May 7 to May 10, and from June 10 to June 12), while we considered longer periods for both lockdown and after lockdown. These differences in the period of assessment were related to the different epidemiological patterns of hospitalized patients or positive contagions in Norway, France, and Italy. For example, the study performed in Norway was conducted during a period (June–September) with a low number of hospitalized COVID-19 patients, not only compared to the peak of the first wave (e.g., in June 2020 the number of patients was 3 compared to 325 people in April 2020), but also, and more importantly, in comparison with the Italian context (e.g., in June 2020 the number of people with COVID-19 symptoms was about 20,000, see Section 4.1). Although we could exclude any variation in the circadian system due to the lack of circadian typology differences among the three groups, an alternative hypothesis for explaining our data could relate to the seasonal effect on the circadian pacemaker (e.g., [26]). In animal models, for example, a seasonal adjustment of this pacemaker has been reported with an increase in wake duration during longer periods of light and with warmer temperatures (e.g., summer) and a decrease in wake duration during periods with less light and lower temperatures (e.g., winter) [26]. However, it is unclear whether artificial light suppresses [27] or interacts [28] with seasonal variations in circadian mechanisms, considering that artificial light interacts with the circadian system [29]. In addition, in a recent study [30], the strongest seasonal effect on wake time and sleep duration was observed during spring (sleep duration decreased compared to winter), and in our study, the spring season covered the lockdown period almost perfectly. Finally, in line with what has been suggested in other studies [7,8], lower subjective sleep and wake quality in the Lockdown and After-Lockdown groups could also depend on the additional effects of the transition into Daylight Saving Time (DST) [31], which in Italy, took place on 29 March 2020.

The results concerning general distress were also unexpected. In fact, we did not find any significant difference between the three groups for the total HADS score, even if, at a descriptive level, we observed a high HADS score for the Lockdown group compared to the other two groups, which reported similar mean scores. A possible explanation for this finding could be related to the fact that for each group, we recruited participants with a tendency to experience general distress. Indeed, it has been reported that the clinical threshold scores for anxiety and depression, using the HADS questionnaire, are equal to 11 [32] and, thus, the total score of 22 observed in our Pre-Lockdown and After-Lockdown groups, as shown in Table 1, indicates that our participants reported high distress at the moment of the survey. Thus, the lack of a significant difference between the groups, as far as the total HADS score is concerned, could depend on a ceiling-like effect. Furthermore, anxiety and depression showed different patterns. In particular, we found higher anxiety levels in the After-Lockdown group than in the Pre-Lockdown group, whereas we found lower levels of depression in the Pre-Lockdown and After-Lockdown groups than in the Lockdown group. The After-Lockdown group corresponded with the sample recruited from June 11 to August 22, 2020, that is, “Phase 3”, implemented by the Italian Government (see Section 4.1), in which there was a complete restart of all business and social activities, and all restrictions regarding movement within and outside Italy were removed. On the one hand, this Italian Phase 3 could explain the lower depression scores in the After-Lockdown group, since it brought the end of home confinement and social distancing. On the other hand, during Phase 3 the number of contagions ranged from 25,909 to 17,503 (see, https://www.rainews.it/ran24/special/2020/covid19/, accessed on 29 January 2022) and, thus, the risk of COVID-19 contagion was still high, which could explain the high anxiety levels in the After-Lockdown group.

The present study has its limitations. First, the cross-sectional nature of the study did not allow us to assess longitudinal changes in the measured variables in the same individuals, which limits any causal inference. However, the fact that in different participants, tested in different periods relative to the first pandemic wave, we found the same path model that confirms that proposed by Mirolli et al. [5], through a longitudinal study, strongly suggests the reliability of the model. Future studies should confirm these findings with other methodological designs. Another limit of the present work is related to the questionnaire used to assess mindfulness. Even if the FFMQ is the most widely used tool for measuring mindfulness [33], the different ways of assessing acceptance (through the two sub-scales of nonjudging and nonreacting) may confound the assessment of this crucial variable. The fact that we confirmed a previous study [5] using the same questionnaire, contributes to a convergent validity of our results, but we recommend further studies using the Philadelphia Mindfulness Scale (PHLMS [34]), which contains a single scale with which acceptance is assessed. Third, our participants filled in only self-report questionnaires, which could limit the reliability and validity of our findings. Although we administered questionnaires with well-established psychometric properties (see Section 4.2), future studies could adopt more objective measures to assess the variables. Finally, our samples were not completely balanced in terms of gender, as there were significantly more females in the Lockdown and After-Lockdown groups than in the Pre-Lockdown group (see Section 4.1). Although we controlled for gender in all analyses, it has been widely documented in psychiatric epidemiology that women are significantly more likely to develop an anxiety disorder throughout the lifespan than men [35]. In a similar way, Liu et al. [36] reported, during the first contagion wave in China, a higher prevalence of Post-Traumatic Stress Symptoms (PTSS) in females, who suffered more re-experiencing [37], negative alterations in cognition or mood [38], and hyperarousal [39]. These symptoms could explain the mixed results, especially those found in the After-Lockdown group. Future studies with a more balanced distribution of women and men in the samples are needed.

## 4. Materials and Methods

### 4.1. Participants and Procedure

We enrolled a total of 250 volunteer participants from the general population (all participants lived in the south of Italy), through email and social media (no mindfulness practice nor any particular interest in mindfulness was required for participation). We administered the same questionnaires (see below) in three different samples during three periods around the first Italian COVID-19 lockdown. Lockdown in Italy started on 9 March 2020, which defined the transition between the Pre-Lockdown and the Lockdown period. However, the end of lockdown was less clear given that in Italy the release of restrictions involved two steps: so-called “Phase 2”, from 4 May to 11 June 2020, in which only some activities could re-open and several restrictions remained active, such as social distancing and limitations to freedom of movement, and the so-called “Phase 3”, from 11 June 2020, in which all activities restarted, including cinemas, discos, and sports, and all restrictions to movement inside and outside Italy were removed. Due to the many limitations maintained during Phase 2, we decided to consider only Phase 3 as our After-Lockdown period, while we included Phase 2 in our Lockdown phase. The Pre-Lockdown group involved 69 participants (29 females, 40 males; mean age = 33.04 years, SD = 12.94; mean education level = 14.84 years, SD = 2.70), who were assessed from 20 December 2019 to 8 March 2020; the Lockdown group included 78 participants (59 females, 19 males; mean age = 29.17 years, SD = 8.50; mean education level = 15.71 years, SD = 2.37), assessed from 10 March to 10 June 2020; the After-Lockdown group recruited 103 participants (86 females, 17 males; mean age = 30.29 years, SD = 9.46; mean education level = 15.15 years, SD = 2.48), assessed from 11 June to 22 August 2020. The Pre-Lockdown group includes 39 participants who were also included in the longitudinal analysis reported in [5]. Overall, the three groups did not differ in age (*F*(2,247) = 2.75, *p* = 0.07) nor in education level (*F*(1,247) = 2.28, *p* = 0.10). On the contrary, the three groups significantly differed in the distribution of females and males (*χ^2^*(2) = 36, *p* < 0.05), with a higher percentage of males in the Pre-Lockdown group than in the Lockdown and After-Lockdown groups. In all three groups, occupational status included unemployment, students, and workers in both private and public fields.

The study protocol was approved by the Ethics Committee of the Department of Psychology at the University of Campania Luigi Vanvitelli and it originally aimed to investigate the relationship between dispositional mindfulness, sleep, and distress. Except for questionnaires administered during the pre-lockdown period (individual administration through paper-and-pencil), all questionnaires were administered online (using the Google Moduli platform).

### 4.2. Materials

The Italian version of the Five Facets Mindfulness Questionnaire (FFMQ [33]) was used to measure dispositional mindfulness. This questionnaire contains 39 items divided into 5 subscales: observing (e.g., “When I’m walking, I deliberately notice the sensations of my body moving”), describing (e.g., “I’m good at finding words to describe my feelings”), acting with awareness (e.g., “When I do things, my mind wanders off and I’m easily distracted”), nonjudging (e.g., “I criticize myself for having irrational or inappropriate emotions”), and nonreacting (e.g., “I perceive my feelings and emotions without having to react to them”). Observing reflects the ability to observe, notice, or attend to the world. Describing refers to the ability to describe experiences of one’s inner world. Acting with awareness indicates the tendency to pay attention to what one is doing. Nonjudging reflects the extent to which one accepts feelings and thoughts without judging one’s inner experience. Finally, nonreacting reflects the tendency to notice emotions and thoughts without reacting to them [33]. For each item, participants expressed their choice on a 5-point Likert scale. Higher total scores indicate higher dispositional mindfulness. According to MAT theory [16], the monitoring component of mindfulness was defined as the observing score, while the acceptance component of mindfulness was defined as the sum score of nonjudging and nonreacting. The psychometric properties of this scale are good [33].

General distress was measured using the Italian version of the Hospital Anxiety and Depression Scale (HADS [32]), consisting of 14 items divided into 2 subscales: anxiety and depression. Using a 4-point scale, participants rated how they had been feeling in the past week. The HADS has demonstrated good psychometric properties [32], and the total score is used to define general psychological distress [32].

The Italian version of the Mini Sleep Questionnaire (MSQ [40]) was administered to detect sleep and wake disorders. In this questionnaire, 6 items measure sleep quality, while the remaining 4 items measure wake quality. In the MSQ, participants are requested to indicate, for each statement, the frequency of occurrence in the past week on a 7-point scale. The subscales for sleep problems and for wake problems were calculated respectively, with higher scores corresponding to more sleep–wake problems. A total MSQ score was calculated by summing the sleep and wake scores. The psychometric properties of the MSQ are good [25].

Taking into account the relationship between circadian typology and sleep problems and/or mental health [41], in order to control for chronotype we administered the Italian version of the reduced Morningness-Eveningness Questionnaire (rMEQ [42]), which has good psychometric properties. The rMEQ consists of 5 items, covering open and closed response format, and the total score is obtained by summing up all the items. Higher scores reflect a morningness preference.

### 4.3. Data Analysis

All data are available in the Supplementary Materials. First, we evaluated the effect of the lockdown phases on the measured variables. To this aim, we conducted a series of one-way between-group ANCOVAs, with group as the independent variable on three levels (Pre-Lockdown, Lockdown, and After-Lockdown), and sex, age, and education level as covariates. We fixed the α level at *p* = 0.05 and a significant main effect was further investigated by means of Tukey-corrected post-hoc analysis. For the sake of clarity, only significant post-hoc contrasts were reported.

Then, we tested the reliability of the model provided by Mirolli et al. [5] in all the different tested groups. Considering both the model in [5] and the ANCOVA results obtained in the present study, we included only the minimum number of variables in the model, in order to obtain interpretable solutions with our sample size. Thus, the model included only three variables: an acceptance score as the predictor, computed as the sum of nonjudging and nonreacting, the anxiety score as the mediator, and the total MSQ score as the outcome (Figure 1). Both sleep and wake subscales of the MSQ showed the same pattern over phases, and thus we decided to only include the total score. Lastly, we controlled for the effect of chronotype (rMEQ score) on MSQ. This model included only six free parameters (acceptance, anxiety, MSQ, rMEQ, gender, and age); given that our smallest sample included 69 participants, all our results are interpretable according to the rule of thumb that requires a 10:1 ratio between observations and free parameters [43]. To assess measurement invariance throughout groups, we compared a multi-group model with the same paths but without any constraints, i.e., a configural invariance model compared with a metric invariance model in which we constrained the path coefficients to be equal across groups [44].

Model fitting was evaluated on the basis of the following indexes: *χ^2^* statistics, comparative fit index (CFI), Tucker–Lewis index (TLI), root mean square error of approximation (RMSEA) with related 90% confidence intervals (CI_RMSEA_), and standard root mean square residual (SRMR). Model fit was considered adequate if it yielded the following values: non-significant *χ^2^*, CFI and TLI above 0.95, RMSEA equal to 0.06 or less, SRMR equal to 0.08 or less [45]. We tested all the direct and indirect paths considered by means of a path analysis model with parameters estimated on 5000 bootstrapped samples. In particular, we used bias-corrected bootstrapped confidence intervals to test the indirect effects from acceptance to sleep–wake problems through anxiety. We conducted all the reported analyses with the lavaan package [46] and R version 4.1.2 [47]. Raw data are available in the Supplementary Materials.

## 5. Conclusions

The present cross-sectional study confirmed that the COVID-19 lockdown negatively affected psychological variables and sleep–wake quality [3,5,6,7,8,9]. In addition, it confirmed the previously proposed model [5], according to which, the acceptance component of mindfulness has a positive effect on sleep–wake quality through the mediating role of (decreased) anxiety. These findings provide further evidence regarding the mechanisms through which mindfulness benefits sleep quality. In addition, they support the use of mindfulness-based interventions (possibly focused on acceptance) for preventing and/or treating insomnia and sleep-related disorders, especially in highly stressful situations, such as the COVID-19 lockdown.

## Figures and Tables

**Table 1:** Mean, standard deviation, ANCOVA statistics and Tukey post-hoc comparisons for the psychological variables measured in the three phases. Significant results are shown in bold.

	Pre-Lockdown (N = 69)		Lockdown (N = 85)		After-Lockdown (N = 103)		One-Way ANCOVA				Tukey Post-hoc		
Variable	M	SD	M	SD	M	SD	SS	MS	F	*p*	PL-L	PL-AL	L-AL
**Observing**	**25.51**	**6.71**	**28.05**	**5.81**	**26.54**	**5.01**	**243**	**121.5**	**3.71**	**<0.05**	**<0.05**	**0.47**	**0.19**
Describing	27.93	6.04	27.53	5.71	27.23	6.4	20	10.0	0.28	0.75	-	-	-
**Actaware**	**27.65**	**5.97**	**26.67**	**5.37**	**25.38**	**5.94**	**221**	**110.6**	**3.39**	**<0.05**	**0.55**	**<0.05**	**0.29**
**Nonjudging**	**27.12**	**6.31**	**25.01**	**6.49**	**24.03**	**6.12**	**397**	**199**	**5.22**	**<0.01**	**<0.10**	**<0.01**	**0.54**
**Nonreacting**	**20.75**	**4.78**	**20.37**	**3.64**	**19.14**	**3.78**	**127**	**63.6**	**4.00**	**<0.05**	**0.83**	**<0.05**	**<0.10**
**Acceptance**	**47.87**	**8.46**	**45.38**	**7.93**	**43.17**	**7.76**	**920**	**460**	**7.68**	**<0.01**	**0.13**	**<0.01**	**0.39**
HADS tot	22.71	4.04	23.73	3.56	22.82	3.76	55	24.8	1.80	0.17	-	-	-
**Anxiety**	**9.55**	**3.16**	**9.86**	**3.24**	**10.78**	**3.3**	**72**	**35.9**	**3.48**	**<0.05**	**0.84**	**<0.05**	**0.14**
**Depression**	**13.16**	**3.1**	**13.87**	**2.69**	**12.04**	**2.46**	**155**	**77.3**	**10.56**	**<0.01**	**0.25**	**<0.05**	**<0.01**
**Sleep**	**14.35**	**6.09**	**16.94**	**6.4**	**19.36**	**5.89**	**1047**	**523**	**14.16**	**<0.01**	**<0.05**	**<0.01**	**<0.05**
**Wake**	**12.91**	**4.76**	**14.95**	**4.59**	**17.05**	**4.46**	**717**	**359**	**18.33**	**<0.01**	**<0.05**	**<0.01**	**<0.01**
**MSQ tot**	**27.26**	**9.54**	**31.88**	**9.8**	**36.41**	**9.03**	**3496**	**1748**	**20.61**	**<0.01**	**<0.01**	**<0.01**	**<0.01**
rMEQ	14.75	3.46	14.64	3.61	13.68	3.91	63	31.4	2.34	0.10	-	-	-

Note: SS = sum of squares, MS = mean square. One-way ANCOVA has 2 degrees of freedom and controlled for the effects of age, sex, and education level. Tukey post-hoc tests are reported only for significant one-way ANCOVAs. PL = Pre-Lockdown, L = Lockdown, and AL = After-Lockdown. Actaware = acting with awareness. Acceptance was calculated as the sum of the Nonjudging and Nonreacting scores.

**Table 2:** SEM estimated coefficients for the configural invariance model.

	Pre-Lockdown Group					Lockdown Group					After-Lockdown Group				
Parameter	b	CI_lower_	CI_upper_	SE	β	b	CI_lower_	CI_upper_	SE	β	b	CI_lower_	CI_upper_	SE	β
**(a)Acceptance->Anxiety**	−0.22 **	−0.30	−0.15	0.04	−0.60	−0.24 **	−0.31	−0.17	0.04	−0.60	−0.23 **	−0.28	−0.16	0.03	−0.53
**(b)Anxiety->Sleep–wake**	0.89 *	0.15	1.74	0.40	0.30	1.35 **	0.60	2.04	0.36	0.45	0.41 +	−0.05	0.94	0.25	0.15
**(c)Acceptance->Sleep–wake**	−0.11	−0.42	0.29	0.18	−0.10	−0.06	−0.33	0.25	0.15	−0.05	−0.57 **	−0.79	−0.36	0.11	−0.49
Indirect effect (a * b)	−0.20 *	−0.44	−0.03	0.10	−0.18	−0.33 **	−0.56	−0.14	0.10	−0.27	−0.09+	−0.23	0.01	0.06	−0.08
**Total effect**	−0.31 *	−0.56	−0.02	0.14	−0.28	−0.38 **	−0.61	−0.16	0.12	−0.31	−0.66 **	−0.84	−0.47	0.09	−0.56

Note: b = unstandardized coefficient, CI_lower_ and CI_upper_ = lower and upper 95% bootstrapped confidence intervals of b, SE = standard error, β = standardized coefficient. Significance level is indicated as follows: + *p* < 0.10, * *p* < 0.05, ** *p* < 0.01.

**Table 3:** Estimated coefficients for the metric invariance model and path c estimated in each phase in the model with the released parameter.

	Metric Invariance Model				
Parameter	b	CI_lower_	CI_upper_	SE	β
**(a)Acceptance- > Anxiety**	−0.23 **	−0.27	−0.19	0.02	−0.61
**(b)Anxiety- > Sleep–wake**	0.76 **	0.38	1.17	0.20	0.24
**(c)Acceptance- > Sleep–wake**	−0.32 **	−0.49	−0.15	0.08	−0.27
Indirect effect (a * b)	−0.17 **	−0.28	−0.08	0.05	−0.15
**Total effect**	−0.50 **	−0.63	−0.36	0.07	−0.42
	**Metric invariance model with released parameter c**				
**Parameter c by phase**	**b**	**CI_lower_**	**CI_upper_**	**SE**	**β**
**Pre-Lockdown group**	−0.16	−0.44	0.18	0.16	−0.14
**Lockdown group**	−0.22 +	−0.46	0.02	0.12	−0.19
**After-Lockdown group**	−0.49 **	−0.69	−0.29	0.10	−0.41

Note: b = unstandardized coefficient, CI_lower_ and CI_upper_ = lower and upper 95% bootstrapped confidence intervals of b, SE = standard error, β = standardized coefficient. Significance level is indicated as follows: + *p* < 0.10, * *p* < 0.05, ** *p* < 0.01.

## Data Availability

The data presented in this study are available in Table S1.

## Supplementary Materials

The dataset is available online at: https://www.mdpi.com/article/10.3390/clockssleep4010016/s1, Table S1: DATA.
